# Lower redemption of monthly Special Supplemental Nutrition Program for Women, Infants and Children benefits associated with higher risk of program discontinuation

**DOI:** 10.1017/S136898002300201X

**Published:** 2023-12

**Authors:** Christopher E Anderson, Catherine E Yepez, Shannon E Whaley

**Affiliations:** Public Health Foundation Enterprises (PHFE) WIC, A Program of Heluna Health, 13181 Crossroads Pkwy N #540, City of Industry, CA 91746, USA

**Keywords:** WIC, Safety net program, Benefit redemption, Food package, Child

## Abstract

**Objective:**

To determine whether Special Supplemental Nutrition Program for Women, Infants, and Children (WIC) food benefit redemption is associated with discontinuing WIC participation, failure to recertify, overall and by race/ethnicity-language preference and participant category.

**Design:**

Retrospective cohort study, using multivariable modified Poisson regression to determine risk ratios (RR) and 95 % CI for associations between household-level food benefit redemption (interval-scaled benefit redemption percent, averaged across WIC benefit subcategories, for the final 3, 6 and 12 months of certification) and failure to recertify in WIC, overall and within strata of race/ethnicity-language preference and participant category.

**Setting:**

WIC administrative data collected November 2019–July 2021 in Southern California.

**Participants:**

WIC-participating children ages 0–3 years at initial certification from November 2019 to May 2020 (*n* 41 263).

**Results:**

In all time periods, and for all subgroups, every 10 % lower food benefit redemption was associated with increased risk of failure to recertify. Among households without missing food benefit data, failure to recertify risk peaked at 505 % higher (RR = 6·05, 95 % CI (5·63, 6·51)) in households with average 12-month redemption <10 % compared with households with ≥70 % redemption.

**Conclusions:**

Lower WIC benefit redemption was associated with higher risk of failing to recertify among participants. Focused nutrition education around benefit redemption may improve WIC retention and child health through incremental increases in food benefit redemption.

The Special Supplemental Nutrition Program for Women, Infants, and Children **(**WIC) is a nutrition assistance program of the federal government of the USA and provides services to pregnant and postpartum women, in addition to infants and children under 5 years of age, who reside households with income at or below 185 % of the federal poverty level and who are at nutritional risk^(1)^. WIC serves over 6 million participants every month^(2)^, reaching 57 % of eligible individuals^(3)^, though participation has declined since 2010^(4)^. The four core services WIC provides are supplemental healthy foods, referred to as the WIC food package, nutrition education and individualised nutrition counseling, breast-feeding support and health and social service referrals^(1)^. WIC food packages were revised to be aligned with the Dietary Guidelines for Americans in 2009^(5)^ and are tailored to support the nutritional needs of children as they age, special dietary considerations and, in infancy, the amount of breast-feeding that a mother reports^(6)^.

Child diet quality improved among WIC-participating children following the food package revisions in 2009^(7,8)^, and WIC foods contribute to improved diet quality among participating children^(9)^. Another substantial change to WIC is the widespread adoption of Electronic Benefit Transfer (EBT) systems in lieu of paper vouchers used for the purchase of WIC foods at approved vendors. As of January 2023, all states and territories have fully transitioned to EBT issuance of WIC benefits^(10)^. This transition brought changes to the WIC benefit redemption experience^(11)^, effectively removing the ‘shopping list’ printed on the WIC paper vouchers and requiring participants to check their remaining WIC food balances either at the vendor (e.g. a grocery store), the WIC site or, in some states, a companion WIC App for the participant’s cellular telephone. The transition to EBT also allows the assessment of the specific food benefits each family redeems monthly^(12,13)^. This change enabled, for the first time, a detailed examination of benefit redemption patterns and how redemption may be associated with ongoing participation in the program.

Longer duration of WIC participation is associated with higher diet quality at age 5 years^(14)^. However, many families do not continue WIC participation for the duration of child program eligibility to age 5, despite continued income eligibility^(15)^. Low benefit utilisation, based upon the redemption of paper vouchers with <75 % compared with ≥75 % redeemed, was associated with lower rates of recertification prior to the introduction of EBT in California^(16)^ and qualitatively in Massachusetts^(17)^. Therefore, it is important to examine factors that may indicate reduced engagement with the program in order to maximise ongoing participation and extend the positive impacts of WIC on child well-being^(14,16–19)^. WIC food benefit redemption data are a new source of information available to WIC agencies in California on participating households since the advent of EBT benefit issuance in 2019. Benefit redemption data might be used to inform educational efforts for WIC participants about how to maximise their use of program services, in addition to assisting WIC program efforts to identify and support families at greater risk of dropping off the program. This study aims to summarise household WIC food benefit redemption over the duration of child WIC certification periods (12 months), to determine whether benefit redemption is associated with the risk of failure to recertify on WIC and to assess whether these relationships vary by participant category or race/ethnicity-language preference. It was hypothesised that higher benefit redemption would be associated with lower risk of failure to recertify, and that this would be observed across all race/ethnicity-language preference and participant category groups.

## Methods

### Data source and study sample

WIC administrative data collected by Public Health Foundation Enterprises WIC, the largest local agency WIC program in the USA, in Southern California were used in this study. Children ages 0 to 3 years who were initially enrolled at a Public Health Foundation Enterprises WIC site that had transitioned to EBT benefit issuance were included in this study. Participant categories for children age 4 years and women (pregnant, postpartum and breast-feeding) were excluded due to either ineligibility for recertification (children age 4 years, postpartum and breast-feeding women) or a distinct risk profile for program discontinuation (pregnancy loss among pregnant women participants). In California, where this study took place, the transition from food benefits issued on paper vouchers to EBT cards happened in 2019^(13)^. Initial certifications between 11 November 2019 and 31 May 2020 (*n* 61 835) were included to ensure that the initial certification period ended before the introduction of an enhanced WIC Cash Value Benefit for fruits and vegetables on 1 June 2021. Issuance and redemption data were collected through July 2021. To ensure that there was sufficient redemption to calculate average values over the study period, children missing issuance data in the first month of the certification period were excluded from the analysis (*n*, excluded = 18 928). Children with excess missing issuance data (missing > 4 months of issuance data in the entire certification period, > 2 months in the last 6 months of the certification period, or >1 month in the last 3 months of the certification period) were excluded from the analysis (*n*, excluded = 1644).

WIC administrative data, collected during routine WIC service administration, contain the dates of the beginning and end of each certification period, child age, race/ethnicity and food package issued each month; caregiver language preference and educational attainment (highest grade completed by the family representative – generally the child’s mother) and household size, number of WIC participating individuals, income, Supplemental Nutrition Assistance Program (SNAP) participation and Medicaid participation. Since November 2019, monthly WIC food benefit redemption data were extracted for households issued food benefits to the WIC EBT card for Public Health Foundation Enterprises WIC families. WIC food benefit redemption data are available only at the household level because they are issued to a family representative and redeemed through a single EBT card, so redemption represents the aggregate redemption across all food benefits issued to every WIC-participating individual in the household.

### Outcome

Ongoing participation in WIC is measured by eligibility recertification in the program every 12 months. Failure to recertify following the index 12-month certification period (beginning between 11 November 2019 and 31 May 2020) was the outcome of interest in this study. In this analysis failure to recertify was defined dichotomously by the issuance of a food package in the 2 months following the expiration of the index certification period. No issuance for the study child within 2 months of the expiration of the index certification qualified as a failure to recertify. Any issuance for the study participating child within 2 months of the expiration indicated a successful recertification.

### Exposure

Household WIC food benefit redemption was the primary exposure of interest for this study. WIC food benefits are issued and redeemed in subcategories, including but not limited to milk, cheese, eggs, cereal, 100 % juice, a cash value benefit for fruits and vegetables, legumes and infant formula. A family’s subcategory specific redemption percentage was calculated each month by dividing the amount redeemed by the amount issued (number of items redeemed divided by the number issued, or for the cash value benefit the United States Dollars redeemed divided by the United States dollars issued). An overall percent redemption was then calculated for each family by calculating the average across all food benefit subcategories that the family was issued that month. Overall percent redemption was then summarised across all months with non-missing issuance for three different time periods of the index certification period. These three periods were the last 12 months, the last 6 months and the last 3 months of the index certification. For regression analyses, redemption was interval scaled in 10 % increments and included in models as a categorical variable.

### Covariates

Covariates available for this analysis included start and end dates of the index certification period for each child participant, participant age and category at the initial certification (0–11 months of age fully formula feeding, 0–11 months of age partially breast-feeding, 0–11 months of age fully breast-feeding, child 1 year of age, child 2 years of age, child 3 years of age), child race/ethnicity and caregiver language preference (non-Hispanic Asian, non-Hispanic Black, Hispanic Spanish-speaking, Hispanic English-speaking, non-Hispanic White and non-Hispanic Other); family representative’s educational attainment (completed less than high school, completed high school and completed more than high school) and household size (continuous), number of household WIC participants in the month before the end of the certification period (continuous), income (continuous, percentage of federal poverty level), Medicaid participation (yes, no) and SNAP participation (yes, no). Variables identified as potential effect modifiers included missing issuance (any, none) during the period of exposure (last 12, 6 or 3 months of the certification period), race/ethnicity-language preference and participant category at the initial certification. WIC service dates, certification period and participant category were determined by WIC-program systems; all other covariates were reported by the child’s caregiver.

### Statistical analysis

Child, caregiver and household characteristics of study participants were summarised with frequencies and percentages or means and standard deviations in eight categories of interval-scaled average redemption over the entire index certification period (<10 %, 10 to <20 %, 20 to <30 %, 30 to <40 %, 40 to <50 %, 50 to <60 %, 60 % to <70 % and 70 % to 100 % redemption). Differences were tested with *χ* ^2^ tests of independence and ANOVA *F*-tests for categorical and continuous variables, respectively.

Risk ratios (RR) and 95 % CI for the association of the exposure of interest, interval scaled average benefit redemption for the last 12 months, the last 6 months and the last 3 months of the certification period, with the outcome, failure to recertify, were calculated with modified Poisson regression models with robust SE estimates, accommodating clustering of children within families^(20)^. No significant differences were identified between average redemption categories above 70 %, so these categories were combined into a single 70 % to 100 % category. Models included independent terms for variables including redemption category, child sex, race/ethnicity-language preference, participant category, family representative educational attainment, household size, number of WIC participants, income, SNAP participation, Medicaid participation and a two-way interaction between redemption and missing issuance to allow estimation of associations in issuance strata (no missing, any missing).

Effect modification by race/ethnicity and participant category were evaluated in separate Poisson regression models, with independent terms for redemption category (last 12 months of certification period), child sex, race/ethnicity-language preference, participant category, family representative educational attainment, household size, number of WIC participants, income, SNAP participation, Medicaid participation, any missing issuance (dichotomous) and a two-way interaction between redemption and either race/ethnicity-language preference or participant category. All analyses were conducted with SAS 9.4 (SAS Institute Inc.). *P* values <0·05 were considered statistically significant.

## Results

Over one-half (55·1 %) of children in this study lived in households that redeemed an average of 70 % to 100 % of their WIC food benefits for the full 12-month certification period (Table 1). Average benefit redemption differed significantly by race/ethnicity-language preference, with children in the highest redemption category (70 % to 100 % redemption) more likely to be non-Hispanic Asian and Hispanic Spanish-speaking and children in the lower redemption categories (<70 % redemption) more likely to Hispanic English-speaking and non-Hispanic Black. Benefit redemption was distributed similarly across participant categories, with small magnitude but statistically significant differences between participant categories observed. Failure to recertify decreased from 57·3 % among those with low (<10 %) redemption to 7·1 % among those with high (70 % to 100 %) redemption. Children from households with higher redemption (70 % to 100 %) were more likely to have a family representative with less than a high school education, a higher household income (≥50 % federal poverty level), more household members (≥4 people) and more household WIC participants (≥2 WIC participants). Children from households with lower redemption (<70 %) were more likely to participate in SNAP.

**Table 1:** Sample characteristics for child WIC participants 0 to 3 years of age at initial certification in Southern California in November 2019 to May 2020 (*n* 41 263)

Variable	Average monthly family food benefit redemption																*P*-value
	<10 %		10 to <20 %		20 to <30 %		30 to <40 %		40 to <50 %		50 to <60 %		60 to <70 %		70 to 100 %		
	*n* 1455		*n* 1323		*n* 1763		*n* 2280		*n* 3036		*n* 3946		*n* 4722		*n* 22 738		
	*n*	%	*n*	%	*n*	%	*n*	%	*n*	%	*n*	%	*n*	%	*n*	%	
Child																	
Failed to recertify	833	57·3	537	40·6	612	34·7	596	26·1	611	20·1	601	15·2	605	12·8	1618	7·1	<0·0001
Male	755	51·9	618	46·7	913	51·8	1177	51·6	1549	51·0	1985	50·3	2362	50·0	11 511	50·6	0·10
Race/ethnicity-language preference																	<0·0001
non-Hispanic Asian	72	4·9	45	3·4	72	4·1	78	3·4	137	4·5	155	3·9	225	4·8	1920	8·4	
non-Hispanic Black	212	14·6	205	15·5	241	13·7	258	11·3	265	8·7	303	7·7	270	5·7	692	3·0	
Hispanic, SP	171	11·8	153	11·6	207	11·7	296	13·0	436	14·4	711	18·0	1013	21·5	8445	37·1	
Hispanic, EN	873	60·0	804	60·8	1071	60·7	1463	64·2	1978	65·2	2499	63·3	2917	61·8	10 386	45·7	
non-Hispanic White	66	4·5	51	3·9	80	4·5	89	3·9	110	3·6	123	3·1	150	3·2	594	2·6	
non-Hispanic Other	61	4·2	65	4·9	92	5·2	96	4·2	110	3·6	155	3·9	147	3·1	701	3·1	
Participant category																	<0·0001
Formula fed infant	56	3·8	62	4·7	129	7·3	245	10·7	333	11·0	499	12·6	577	12·2	2412	10·6	
Partially breastfed infant	33	2·3	39	2·9	59	3·3	92	4·0	187	6·2	256	6·5	344	7·3	1804	7·9	
Fully breastfed infant	48	3·3	39	2·9	74	4·2	90	3·9	117	3·9	161	4·1	180	3·8	788	3·5	
Child 1–2 years	606	41·6	522	39·5	654	37·1	759	33·3	949	31·3	1222	31·0	1414	29·9	6413	28·2	
Child 2–3 years	414	28·5	359	27·1	477	27·1	600	26·3	783	25·8	953	24·2	1137	24·1	5775	25·4	
Child 3–4 years	298	20·5	302	22·8	370	21·0	494	21·7	667	22·0	855	21·7	1070	22·7	5546	24·4	
Caregiver/household																	
≥1 month missing issuance in the																	
Last 12 months	174	12·0	119	9·0	156	8·8	178	7·8	211	6·9	248	6·3	254	5·4	777	3·4	<0·0001
Last 6 months	73	5·0	57	4·3	80	4·5	85	3·7	99	3·3	122	3·1	106	2·2	357	1·6	<0·0001
Last 3 months	61	4·2	44	3·3	70	4·0	72	3·2	76	2·5	103	2·6	96	2·0	304	1·3	<0·0001
Family representative education																	<0·0001
<High school	369	25·4	308	23·3	393	22·3	540	23·8	740	24·4	940	23·8	1261	26·8	7035	31·0	
Completed high school	723	49·9	651	49·2	862	48·9	1089	47·9	1460	48·2	1901	48·2	2171	46·1	9332	41·1	
>High school	358	24·7	363	27·5	506	28·7	644	28·3	830	27·4	1101	27·9	1282	27·2	6330	27·9	
Medicaid	1063	73·1	963	72·8	1240	70·3	1627	71·4	2078	68·4	2703	68·5	3234	68·5	15 811	69·5	0·0007
SNAP	522	35·9	490	37·0	571	32·4	729	32·0	872	28·7	1035	26·2	1215	25·7	5063	22·3	<0·0001
Household income																	<0·0001
<50 % FPL	584	40·1	532	40·2	628	35·6	786	34·5	1016	33·5	1213	30·7	1374	29·1	5438	23·9	
50 to 100 % FPL	486	33·4	453	34·2	622	35·3	841	36·9	1131	37·3	1521	38·5	1878	39·8	9679	42·6	
>100 % FPL	385	26·5	338	25·5	513	29·1	653	28·6	889	29·3	1212	30·7	1470	31·1	7621	33·5	
Household size																	<0·0001
<4 people	758	52·1	713	53·9	843	47·8	1047	45·9	1212	39·9	1590	40·3	1789	37·9	7192	31·6	
4–5 people	556	38·2	502	37·9	773	43·8	952	41·8	1463	48·2	1881	47·7	2320	49·1	12 054	53·0	
≥6 people	141	9·7	108	8·2	147	8·3	281	12·3	361	11·9	475	12·0	613	13·0	3492	15·4	
Family members on WIC																	<0·0001
1	1344	92·4	1140	86·2	1382	78·4	1717	75·3	2219	73·1	2755	69·8	3296	69·8	15 500	68·2	
2	96	6·6	163	12·3	293	16·6	445	19·5	624	20·6	934	23·7	1155	24·5	5820	25·6	
≥3	15	1·0	20	1·5	88	5·0	118	5·2	193	6·4	257	6·5	271	5·7	1418	6·2	

EN, English speaking; FPL, federal poverty level; SNAP, Supplemental Nutrition Assistance Program; SP, Spanish speaking; WIC, Special Supplemental Nutrition Program for Women, Infants, and Children.

Lower average food benefit redemption was associated with higher risk of failure to recertify (Table 2). The risk of failure to recertify increased with each 10 % decrease in interval scaled average food benefit redemption among children with no missing issuance. For children with average redemption of <10 %, 10 to <20 %, 20 to <30 %, 30 to <40 %, 40 to <50 %, 50 to <60 % and 60 to <70 % over the last 12 months of the certification period, the risk of failing to recertify was 505 % higher (RR (95 % CI), 6·05 (5·63, 6·51)), 337 % higher (4·37 (4·00, 4·77)), 310 % higher (4·10 (3·77, 4·47)), 216 % higher (3·16 (2·89, 3·45)), 153 % higher (2·53 (2·32, 2·77)), 96 % higher (1·96 (1·79, 2·15)) and 65 % higher (1·65 (1·50, 1·80)), respectively, compared with those with average redemption between 70 % and 100 %. This stepped increase in risk of failure to recertify for each 10 % decrease in redemption percentage was also observed among those with missing issuance, though associations were of more modest magnitudes. Similar magnitudes of association were observed for average redemption over the last 6 months and the last 3 months of the certification period.

**Table 2:** Association between average family WIC food benefit redemption and child failure to recertify among WIC-participating children 0–3 years of age at initial certification in November 2019 to May 2020 (*n* 41 263)

Redemption category	Last 12 months				Last 6 months				Last 3 months			
	No missing issuance		Any missing issuance		No missing issuance		Any missing issuance		No missing issuance		Any missing issuance	
	RR	95 % CI*	RR	95 % CI*	RR	95 % CI†	RR	95 % CI†	RR	95 % CI‡	RR	95 % CI‡
<10 %	6·05	5·63, 6·51	3·55	2·94, 4·28	6·29	5·90, 6·71	3·22	2·55, 4·07	5·90	5·54, 6·29	3·46	2·69, 4·46
10 to <20 %	4·37	4·00, 4·77	3·28	2·63, 4·10	3·84	3·51, 4·20	2·55	1·87, 3·48	3·32	2·99, 3·70	2·81	2·01, 3·93
20 to <30 %	4·10	3·77, 4·47	2·77	2·22, 3·45	3·37	3·06, 3·70	2·80	2·13, 3·70	2·80	2·54, 3·09	1·66	1·10, 2·50
30 to <40 %	3·16	2·89, 3·45	2·38	1·89, 2·99	2·52	2·29, 2·77	2·35	1·74, 3·17	2·21	2·00, 2·44	2·19	1·57, 3·05
40 to <50 %	2·53	2·32, 2·77	1·81	1·40, 2·34	2·07	1·88, 2·28	1·82	1·33, 2·50	1·98	1·79, 2·19	1·69	1·12, 2·54
50 to <60 %	1·96	1·79, 2·15	1·74	1·36, 2·22	1·68	1·52, 1·86	0·98	0·64, 1·52	1·41	1·26, 1·58	1·75	1·21, 2·51
60 to <70 %	1·65	1·50, 1·80	1·72	1·34, 2·21	1·46	1·32, 1·61	1·21	0·82, 1·78	1·37	1·23, 1·51	0·69	0·38, 1·25
70 to 100 %	1·00	Ref	1·00	Ref	1·00	Ref	1·00	Ref	1·00	Ref	1·00	Ref

FPL, federal poverty level; RR, risk ratio; SNAP, Supplemental Nutrition Assistance Program; WIC, Special Supplemental Nutrition Program for Women, Infants and Children.

* Association is RR (95 % CI) among children with 0 months (*n* 39 146) and >0 months (*n* 2117) of missing issuance during the certification period. Average redemption was based upon all months issued during the full 12-month certification period. The modified Poisson regression model with robust SE estimation accommodated clustering of multiple children within families, including terms for redemption category, any missing issuance and a two-way interaction between redemption category and missing issuance. The model was adjusted for: child sex, race/ethnicity-language preference, category (i.e. age); family representative educational attainment and household size (continuous), number of household WIC recipients (continuous), income (continuous, FPL percentage), SNAP participation and Medicaid participation.

† Association is RR (95 % CI) among children with 0 months (*n* 40 284) and >0 months (*n* 979) of missing issuance during the final 6 months of the certification period. Average redemption was based upon all months issued during the final 6 months of the certification period. The modified Poisson regression model with robust SE estimation accommodated clustering of multiple children within families, including terms for redemption category, any missing issuance and a two-way interaction between redemption category and missing issuance. The model was adjusted for: child sex, race/ethnicity-language preference, category (i.e. age); family representative educational attainment and household size (continuous), number of household WIC recipients (continuous), income (continuous, FPL percentage), SNAP participation and Medicaid participation.

‡ Association is RR (95 % CI) among children with 0 months (*n* 40 437) and >0 months (*n* 826) of missing issuance during the final 3 months of the certification period. Average redemption was based upon all months issued during the final 3 months of the certification period. The modified Poisson regression model with robust SE estimation accommodated clustering of multiple children within families, including terms for redemption category, any missing issuance and a two-way interaction between redemption category and missing issuance. The model was adjusted for: child sex, race/ethnicity-language preference and category (i.e. age); family representative educational attainment and household size (continuous), number of household WIC recipients (continuous), income (continuous, FPL percentage), SNAP participation and Medicaid participation.

In the regression model evaluating the relationship of redemption with failure to recertify within race/ethnicity groups, similar patterns of association were observed between each 10 % lower redemption and higher risk of failure to recertify in all race/ethnicity-language preference strata (Table 3). The interaction between race/ethnicity-language preference and redemption was not statistically significant, indicating that the relationship between lower redemption and higher risk of failure to recertify was not subject to effect modification by race/ethnicity-language preference (interaction *P* value = 0·13). When the relationship between redemption and recertification was evaluated within participant categories, the relationship between lower redemption and higher risk of failure to recertify was observed in all groups. Effect modification of the relationship between redemption and failure to recertify by participant category was identified (interaction *P*-value < 0·0001), with significantly smaller magnitudes for associations between lower redemption and failure to recertify observed among children 2 years of age and children 3 years of age relative to infants and children 1 year of age.

**Table 3:** Association between redemption during 12-month certification period and failure to recertify by child race/ethnicity-language preference and participant category (*n* 41 263)

Redemption category	Child race/ethnicity-language preference											
	non-Hispanic Asian *n* 2704		non-Hispanic Black *n* 2446		Hispanic, SP *n* 11 432		Hispanic, EN *n* 21 991		non-Hispanic White *n* 1263		non-Hispanic Other *n* 1426	
	RR	95 % CI*	RR	95 % CI*	RR	95 % CI*	RR	95 % CI*	RR	95 % CI*	RR	95 % CI*
<10 %	5·75	4·29, 7·71	5·21	3·98, 6·80	5·35	4·36, 6·57	5·65	5·18, 6·17	5·79	4·23, 7·93	8·16	5·86, 11·36
10 to <20 %	6·49	4·67, 9·01	3·86	2·88, 5·17	5·06	4·06, 6·32	4·00	3·60, 4·45	4·15	2·81, 6·12	6·75	4·72, 9·65
20 to <30 %	4·37	2·97, 6·42	3·99	3·00, 5·31	4·01	3·18, 5·05	3·76	3·39, 4·16	5·04	3·57, 7·11	4·98	3·43, 7·24
30 to <40 %	4·08	2·81, 5·91	3·21	2·37, 4·34	2·71	2·11, 3·49	2·98	2·68, 3·31	3·45	2·35, 5·07	4·38	2·97, 6·47
40 to <50 %	1·95	1·25, 3·05	2·43	1·74, 3·39	2·34	1·86, 2·96	2·41	2·17, 2·68	2·89	1·95, 4·28	3·93	2·65, 5·83
50 to <60 %	2·13	1·41, 3·21	1·45	1·00, 2·09	2·02	1·64, 2·49	1·91	1·71, 2·13	2·88	1·92, 4·33	2·85	1·87, 4·36
60 to <70 %	2·16	1·51, 3·10	1·97	1·39, 2·78	1·52	1·23, 1·87	1·63	1·46, 1·82	1·30	0·76, 2·23	2·22	1·39, 3·54
70 to 100 %	1·00	Ref	1·00	Ref	1·00	Ref	1·00	Ref	1·00	Ref	1·00	Ref

Child participant category												
Redemption category	Infant, fully formula feeding *n* 4313		Infant, partial breast-feeding *n* 2814		Infant, fully breast-feeding *n* 1497		Child, 1–2 years *n* 12 539		Child, 2–3 years *n* 10 498		Child, 3–4 years *n* 9602	
	RR	95 % CI†	RR	95 % CI†	RR	95 % CI†	RR	95 % CI†	RR	95 % CI†	RR	95 % CI†
<10 %	7·96	6·41, 9·87	8·42	5·29, 13·41	12·29	7·83, 19·28	9·28	8·12, 10·61	6·15	5·41, 7·01	2·88	2·54, 3·26
10 to <20 %	5·76	4·32, 7·68	9·88	6·62, 14·74	9·76	5·88, 16·19	6·52	5·57, 7·62	4·37	3·71, 5·15	2·59	2·25, 2·97
20 to <30 %	4·85	3·77, 6·24	7·37	4·87, 11·15	9·12	5·67, 14·69	6·17	5·28, 7·19	4·05	3·45, 4·76	2·32	2·01, 2·67
30 to <40 %	3·50	2·76, 4·45	5·77	3·79, 8·80	5·61	3·27, 9·63	4·46	3·76, 5·28	3·53	3·00, 4·15	1·87	1·60, 2·17
40 to <50 %	2·80	2·19, 3·59	4·73	3·30, 6·78	3·55	1·93, 6·51	3·56	2·98, 4·24	2·84	2·40, 3·35	1·52	1·31, 1·77
50 to <60 %	2·18	1·71, 2·79	3·37	2·30, 4·94	2·93	1·61, 5·34	2·71	2·26, 3·24	2·16	1·81, 2·57	1·34	1·16, 1·56
60 to <70 %	1·51	1·15, 1·98	2·85	1·96, 4·16	2·11	1·11, 4·01	2·18	1·80, 2·63	2·04	1·72, 2·42	1·21	1·05, 1·40
70 to 100 %	1·00	Ref	1·00	Ref	1·00	Ref	1·00	Ref	1·00	Ref	1·00	Ref

EN, English-speaking; FPL, federal poverty level; RR, risk ratio; SNAP, Supplemental Nutrition Assistance Program; SP, Spanish-speaking; WIC, Special Supplemental Nutrition Program for Women, Infants and Children.

* Association is RR (95 % CI). Average redemption was based upon all months issued during the full 12-month certification period. The modified Poisson regression model with robust SE estimation accommodated clustering of multiple children within families, including terms for redemption category, child race and a two-way interaction between redemption category and child race/ethnicity-language preference. The model was adjusted for: child sex and category (i.e. age); family representative educational attainment and household size (continuous), number of household WIC recipients (continuous), income (continuous, FPL percentage), SNAP participation, Medicaid participation and any missing issuance data (dichotomous).

† Association is RR (95 % CI). Average redemption was based upon all months issued during the full 12-month certification period. The modified Poisson regression model with robust SE estimation accommodated clustering of multiple children within families, including terms for redemption category, child participation category and a two-way interaction between redemption category and child participation category. The model was adjusted for: child sex and race/ethnicity-language preference; family representative educational attainment and household size (continuous), number of household WIC recipients (continuous), income (continuous, FPL percentage), SNAP participation, Medicaid participation and any missing issuance data (dichotomous).

## Discussion

WIC food benefit redemption below 70 % was associated with higher risk of failure to recertify among WIC participants in Southern California between November 2019 and July 2021, and the risk of failure to recertify increased with each 10 % decrease in food benefit redemption. Lower average WIC food benefit redemption calculated across the last 12 months, the last 6 months and the last 3 months of the index certification period was associated with higher risk of failure to recertify. This association of lower food benefit redemption with higher risk of failure to recertify was observed among children with and without missing issuance data, among children of all race/ethnicity-language preference groups and among children receiving all WIC food packages.

The current study aligns with previously reported associations of both lower food benefit redemption and missing food benefit issuance with lower odds of recertification among WIC participants in Southern California^(16)^ and extends those results to find increased risk of failure to recertify for each 10 % decrease in interval scaled redemption below 70 % among WIC participants of all race/ethnicity groups and of all categories of infant/child participants. A qualitative examination of WIC benefit use and program participation found that benefit utilisation was lower among families who stopped participating in WIC, and that this relationship was driven by perception of lower value, acceptability and appropriateness of WIC food benefits for their children^(17)^.

In regression models that included an interaction between child race/ethnicity-language preference and food benefit redemption, no significant effect modification by race/ethnicity-language preference was identified, and similar patterns of higher risk of failure to recertify with each 10 % decrease in redemption were observed in each race/ethnicity-language preference group. Race/ethnicity-language preference is associated with rates of recertification and duration of WIC participation^(14)^ and with different perception of and preferences regarding the contents of WIC food packages^(21)^. The similarity of the association between redemption and failure to recertify across race/ethnicity-language preference strata in the present study suggests that differences in redemption (non-Hispanic Black participants redeeming a smaller percent of WIC food benefits, Spanish-speaking Hispanic participants redeeming a higher percent of WIC food benefits) may contribute to race/ethnicity patterns in participation^(14)^. Further research is needed to understand factors contributing to observed differences in the distribution of redemption by race/ethnicity-language preference^(22–24)^, which may include availability of culturally appropriate substitutions within the WIC food packages^(21,25)^ and structural barriers to WIC benefit redemption including access to WIC-participating vendors with desired WIC foods^(26–29)^, and whether individual- and vendor-level interventions to increase redemption can reduce disparities in WIC retention.

The current study identified stronger associations between lower redemption and higher risk of failure to recertify among infants and children under 2 years of age compared with children older than 2. Prior studies have reported that incomplete redemption varies by food category and decreases among older children^(12)^. This may be due to lower rates of participation among older children^(15)^, meaning that the risk of failure to recertify is higher for the referent category among older children, leading to more modest relative risk estimates. Alternatively, as the contents of WIC food packages change as children age, the different contents of WIC food packages may contribute to differences in redemption and recertification^(6)^. For example, children age 1 year can receive whole fat milk from WIC, while children over age 2 years receive only reduced fat milk^(6)^, and the rate of incomplete redemption was much higher for reduced fat milk than whole fat milk in an analysis of Oklahoma WIC redemption data^(12)^. These prior results suggest that families of older children may identify lower value for the specific contents of child WIC food packages, leading to higher rates of failure to recertify at all levels of food benefit redemption. Finally, a weaker association between redemption and failure to recertify among older children may be due to greater relative value of other program components, including WIC nutrition education, among families of children who remain on the program past age 2 years^(14,30)^.

WIC food packages support the purchase of select healthy foods and beverages among participating households^(6)^. The association between lower redemption of WIC food benefits and higher rates of failure to recertify may be due to lower perceived value of the program among households that redeem less of their benefits^(12,17)^. Alternate explanations for lower benefit redemption include potential difficulties for some participants in accessing WIC approved vendors, though evidence of the association between the food environment and food purchasing and WIC redemption behaviours is mixed^(31,32)^. Participants may also have lower redemption due to difficulty identifying desired WIC-eligible foods at WIC-approved vendors^(11)^, including limited flexibility to accommodate culturally appropriate substitutions^(17)^. Finally, following the transition to EBT cards for WIC benefits, some participants may not remember what foods are WIC eligible and unredeemed in the absence of a printed shopping list or a WIC companion App for a cellular phone^(33)^.

This study has a number of strengths. WIC administrative data collected by the largest local agency WIC program in the USA were used in this study, ensuring a large representative sample with sufficient numbers of participants in all subgroups of interest (race/ethnicity-language preference and WIC participant category). WIC administrative data contain variables that allow characterisation of study participants and statistical adjustment for a number of potential confounders including household socio-economic status. The study period evaluated (November 2019 to July 2021) was chosen to ensure that the increased cash value benefit for fruits and vegetables, which began on 1 June 2021^(34)^, occurred after the expiration of the index certification period so that it would not influence the probability of recertification. The study also has limitations including the occurrence of the COVID-19 pandemic during the study, with concomitant shortages of WIC foods observed in the initial months of the pandemic which may have altered household WIC food benefit redemption^(35)^. However, no differences were observed between analyses that included the early months of the pandemic (last 12 month and last 6 month redemption) and those that did not include the early months of the pandemic (last 3 month redemption), suggesting that WIC food shortages did not bias this study’s reported associations. The observational study design precludes inferring causation. Families who perceive less need for WIC benefits, possibly due to improved socio-economic status, may stop using benefits and not recertify because they no longer perceive a need for WIC support^(17)^. Caution should be exercised when generalising these results to demographically distinct WIC populations from other regions, though the association of lower redemption with higher risk of failure to recertify was observed in all race/ethnicity-language preference groups.

## Conclusions

This study is the first to report an association between redemption of WIC food benefits via EBT and the risk of failure to recertify among child WIC participants ages 0 to 3 years. The association of lower redemption with higher risk of failure to recertify was apparent in all race/ethnicity-language preference groups and among recipients of all WIC infant and child food packages. These results suggest that low redemption may be an important means of identifying participants at risk of leaving the program. Education to provide all WIC participants with integrated information about how to redeem food benefits and how WIC foods can be used in a daily healthy diet are already a cornerstone of WIC services. Results of the current study suggest that efforts to develop and conduct more targeted education with participants exhibiting redemption of less than 70 % of monthly benefits should be evaluated. Given the widespread adoption of EBT, states can also explore how to optimise the use of their Management Information Systems such that front-line staff have immediate access to a family’s redemption history, thereby facilitating targeted education to families exhibiting low redemption. More research is needed to understand patterns of benefit redemption by racial/ethnic composition of the household (i.e. both caregivers and children), across benefit subcategories and longitudinally, to identify how structural barriers contribute to racial/ethnic differences in benefit utilisation, and to understand the associations between redemption of specific WIC foods with program participation and participant outcomes including household food insecurity and child diet. Longer WIC participation has been associated with healthier diets and lower food insecurity rates, and more research identifying factors that may be contributing to early program discontinuation is needed to maximise the programme’s nutritional benefits. The WIC program, in collecting redemption data for all participants following the implementation of EBT for food benefit issuance, has the potential to conduct detailed evaluations of participant benefit redemption data to facilitate program refinements to maximise program participation and the health benefits to participating children.
